# 2-{[4-(Dimethyl­amino)­benzyl­idene]amino}­phenyl disulfide

**DOI:** 10.1107/S1600536811046277

**Published:** 2011-11-09

**Authors:** Qing-Peng He, Li Dai, Bo Tan

**Affiliations:** a College of Chemistry and Chemical Engineering, Liaocheng University, Shandong 252059, People’s Republic of China; b Liaocheng Bureau of Quality and Technical Supervision, Shandong 252059, People’s Republic of China

## Abstract

In the title mol­ecule, C_30_H_30_N_4_S_2_, the two benzene rings connected through the disulfide chain form a dihedral angle of 88.7 (1)°, and the two benzene rings in the benzyl­ideneaniline fragments form dihedral angles of 34.0 (1) and 35.4 (1)°. The crystal packing exhibits no significantly short inter­molecular contacts.

## Related literature

For biological activity of Shiff base derivatives, see: Loncle *et al.* (2004 ▶); Li *et al.* (2004 ▶). For a related structure, see: Roy *et al.* (2009 ▶).
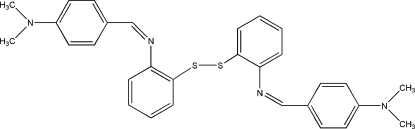


## Experimental

### 

#### Crystal data

C_30_H_30_N_4_S_2_
*M* *_r_* = 510.70
Monoclinic, 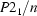

*a* = 10.673 (1) Å
*b* = 22.906 (2) Å
*c* = 11.2939 (11) Å
β = 95.784 (1)°
*V* = 2747.0 (4) Å^3^
*Z* = 4
Mo *K*α radiation
μ = 0.22 mm^−1^
*T* = 298 K
0.30 × 0.26 × 0.12 mm

#### Data collection

Bruker SMART APEX CCD area-etector diffractometer
Absorption correction: multi-scan (*SADABS*; Sheldrick, 1996 ▶) *T*_min_ = 0.937, *T*_max_ = 0.974
13873 measured reflections
4851 independent reflections
2145 reflections with *I* > 2σ(*I*)
*R*_int_ = 0.057

#### Refinement

*R*[*F* ^2^ > 2σ(*F* ^2^)] = 0.051
*wR*(*F* ^2^) = 0.122
*S* = 0.83
4851 reflections
329 parameters
H-atom parameters constrained
Δρ_max_ = 0.29 e Å^−3^
Δρ_min_ = −0.14 e Å^−3^

### 

Data collection: *SMART* (Bruker, 2007 ▶); cell refinement: *SAINT* (Bruker, 2007 ▶); data reduction: *SAINT*; program(s) used to solve structure: *SHELXS97* (Sheldrick, 2008 ▶); program(s) used to refine structure: *SHELXL97* (Sheldrick, 2008 ▶); molecular graphics: *SHELXTL* (Sheldrick, 2008 ▶); software used to prepare material for publication: *SHELXTL*.

## Supplementary Material

Crystal structure: contains datablock(s) I, global. DOI: 10.1107/S1600536811046277/cv5186sup1.cif

Structure factors: contains datablock(s) I. DOI: 10.1107/S1600536811046277/cv5186Isup2.hkl

Supplementary material file. DOI: 10.1107/S1600536811046277/cv5186Isup3.cml

Additional supplementary materials:  crystallographic information; 3D view; checkCIF report

## Figures and Tables

**Fig. 1.**
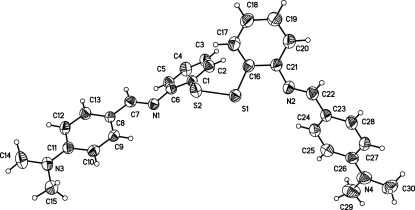
ORTEP drawing of the title complex with atomic numbering scheme and thermal ellipsoids at 30% probability level.

## supplementary crystallographic information

### Comment

In recent years, a number of Schiff base derivatives were shown to exhibit a wide range of interesting biological activities, including antibacteriale antifungal, anticonvulsant, anticancer activities as well as herbicidal and fungicidal activity (Loncle *et al.*, 2004; Li *et al.*, 2004). As a part of our continuing interest in Schiff's bases contained imine N and anionic S atoms, we report here the crystal structure of the title compound (I).

In (I) (Fig. 1), the molecule has a *trans* configuration about the S—S bond. All bond lengths and angles are normal and comparable to those observed in similar compound 2,2'-(disulfanediylbis(2,1-phenylenenitrilomethylylidene)) bis(4,6-di-tbutylphenol) (Roy *et al.*, 2009). Two benzene rings connected through disulfide chain form a dihedral angle of 88.7 (1)°, and two benzene rings in two benzylideneaniline fragments form the dihedral angles of 34.0 (1) and 35.4 (1)°, respectively. The crystal packing exhibits no significantly short intermolecular contacts.

### Experimental

4-(Dimethylamino)benzaldehyde (6 mmol) and 2,2'-diaminodiphenyl disulfide (3 mmol) was refluxed in 20 ml of ethanol for 4.0 h, the mixture then cooling slowly to room temperature and affording the title compound, then recrystallized from ethanol, affording the title compound as a yellow crystalline solid.

### Refinement

All H atoms were positioned geometrically, with C—H=0.93–0.96 Å, and refined as riding, with *U*_iso_(H)=1.2–1.5 *U*_eq_(C).

### Crystal data

**Table:**

C_30_H_30_N_4_S_2_	*F*(000) = 1080
*M**_r_* = 510.70	*D*_x_ = 1.235 Mg m^−^^3^
Monoclinic, *P*2_1_/*n*	Mo *K*α radiation, λ = 0.71073 Å
*a* = 10.673 (1) Å	Cell parameters from 1564 reflections
*b* = 22.906 (2) Å	θ = 2.5–18.1°
*c* = 11.2939 (11) Å	µ = 0.22 mm^−^^1^
β = 95.784 (1)°	*T* = 298 K
*V* = 2747.0 (4) Å^3^	Block, yellow
*Z* = 4	0.30 × 0.26 × 0.12 mm

### Data collection

**Table:**

Bruker SMART APEX CCD area-etector diffractometer	4851 independent reflections
Radiation source: fine-focus sealed tube	2145 reflections with *I* > 2σ(*I*)
graphite	*R*_int_ = 0.057
phi and ω scans	θ_max_ = 25.0°, θ_min_ = 2.5°
Absorption correction: multi-scan (*SADABS*; Sheldrick, 1996)	*h* = −12→12
*T*_min_ = 0.937, *T*_max_ = 0.974	*k* = −27→25
13873 measured reflections	*l* = −13→11

### Refinement

**Table:**

Refinement on *F*^2^	Primary atom site location: structure-invariant direct methods
Least-squares matrix: full	Secondary atom site location: difference Fourier map
*R*[*F*^2^ > 2σ(*F*^2^)] = 0.051	Hydrogen site location: inferred from neighbouring sites
*wR*(*F*^2^) = 0.122	H-atom parameters constrained
*S* = 0.83	*w* = 1/[σ^2^(*F*_o_^2^) + (0.0486*P*)^2^] where *P* = (*F*_o_^2^ + 2*F*_c_^2^)/3
4851 reflections	(Δ/σ)_max_ < 0.001
329 parameters	Δρ_max_ = 0.29 e Å^−^^3^
0 restraints	Δρ_min_ = −0.14 e Å^−^^3^

### Special details

**Table:**

Geometry. All e.s.d.'s (except the e.s.d. in the dihedral angle between two l.s. planes) are estimated using the full covariance matrix. The cell e.s.d.'s are taken into account individually in the estimation of e.s.d.'s in distances, angles and torsion angles; correlations between e.s.d.'s in cell parameters are only used when they are defined by crystal symmetry. An approximate (isotropic) treatment of cell e.s.d.'s is used for estimating e.s.d.'s involving l.s. planes.
Refinement. Refinement of *F*^2^ against ALL reflections. The weighted *R*-factor *wR* and goodness of fit *S* are based on *F*^2^, conventional *R*-factors *R* are based on *F*, with *F* set to zero for negative *F*^2^. The threshold expression of *F*^2^ > σ(*F*^2^) is used only for calculating *R*-factors(gt) *etc*. and is not relevant to the choice of reflections for refinement. *R*-factors based on *F*^2^ are statistically about twice as large as those based on *F*, and *R*- factors based on ALL data will be even larger.

### Fractional atomic coordinates and isotropic or equivalent isotropic displacement parameters (Å^2^)

**Table:**

	*x*	*y*	*z*	*U*_iso_*/*U*_eq_	
S1	0.19747 (8)	0.14806 (4)	0.72993 (7)	0.0713 (3)	
S2	0.11991 (8)	0.22645 (4)	0.67335 (8)	0.0755 (3)	
N1	−0.1215 (2)	0.28105 (12)	0.6062 (2)	0.0628 (7)	
N2	0.3431 (2)	0.04770 (12)	0.7508 (2)	0.0705 (8)	
N3	−0.3191 (3)	0.51151 (12)	0.8653 (3)	0.0786 (8)	
N4	0.1784 (4)	−0.16806 (15)	1.0685 (3)	0.0984 (10)	
C1	0.0171 (3)	0.20663 (13)	0.5451 (3)	0.0584 (8)	
C2	0.0478 (3)	0.16385 (15)	0.4662 (3)	0.0704 (10)	
H2	0.1245	0.1445	0.4793	0.085*	
C3	−0.0350 (4)	0.14966 (15)	0.3680 (3)	0.0805 (10)	
H3	−0.0139	0.1209	0.3155	0.097*	
C4	−0.1484 (4)	0.17819 (16)	0.3485 (3)	0.0841 (11)	
H4	−0.2047	0.1683	0.2834	0.101*	
C5	−0.1786 (3)	0.22166 (15)	0.4258 (3)	0.0745 (10)	
H5	−0.2556	0.2408	0.4122	0.089*	
C6	−0.0955 (3)	0.23708 (14)	0.5236 (3)	0.0582 (8)	
C7	−0.1777 (3)	0.32766 (15)	0.5692 (3)	0.0660 (9)	
H7	−0.1983	0.3318	0.4877	0.079*	
C8	−0.2120 (3)	0.37512 (14)	0.6467 (3)	0.0612 (9)	
C9	−0.1778 (3)	0.37524 (14)	0.7692 (3)	0.0673 (9)	
H9	−0.1314	0.3441	0.8036	0.081*	
C10	−0.2107 (3)	0.42006 (15)	0.8401 (3)	0.0700 (9)	
H10	−0.1851	0.4189	0.9213	0.084*	
C11	−0.2818 (3)	0.46755 (14)	0.7935 (3)	0.0631 (9)	
C12	−0.3114 (3)	0.46861 (14)	0.6697 (3)	0.0716 (10)	
H12	−0.3553	0.5002	0.6344	0.086*	
C13	−0.2766 (3)	0.42355 (15)	0.5998 (3)	0.0723 (10)	
H13	−0.2971	0.4257	0.5179	0.087*	
C14	−0.3916 (3)	0.56013 (16)	0.8147 (3)	0.1046 (13)	
H14A	−0.3512	0.5762	0.7498	0.157*	
H14B	−0.3972	0.5895	0.8745	0.157*	
H14C	−0.4746	0.5471	0.7862	0.157*	
C15	−0.3011 (4)	0.50616 (15)	0.9928 (3)	0.1034 (13)	
H15A	−0.3390	0.4706	1.0165	0.155*	
H15B	−0.3396	0.5388	1.0284	0.155*	
H15C	−0.2126	0.5056	1.0187	0.155*	
C16	0.3436 (3)	0.14305 (15)	0.6678 (3)	0.0619 (9)	
C17	0.3972 (4)	0.18706 (15)	0.6046 (3)	0.0798 (10)	
H17	0.3565	0.2228	0.5932	0.096*	
C18	0.5101 (4)	0.17773 (18)	0.5590 (4)	0.0983 (13)	
H18	0.5459	0.2073	0.5171	0.118*	
C19	0.5702 (4)	0.1251 (2)	0.5750 (4)	0.1061 (14)	
H19	0.6458	0.1188	0.5425	0.127*	
C20	0.5192 (4)	0.08124 (17)	0.6390 (3)	0.0945 (12)	
H20	0.5609	0.0457	0.6493	0.113*	
C21	0.4060 (3)	0.08967 (15)	0.6882 (3)	0.0673 (9)	
C22	0.4045 (3)	0.00799 (16)	0.8111 (3)	0.0750 (10)	
H22	0.4919	0.0084	0.8145	0.090*	
C23	0.3452 (3)	−0.03744 (15)	0.8743 (3)	0.0657 (9)	
C24	0.2152 (3)	−0.04379 (15)	0.8688 (3)	0.0710 (10)	
H24	0.1641	−0.0184	0.8214	0.085*	
C25	0.1604 (3)	−0.08633 (16)	0.9311 (3)	0.0768 (10)	
H25	0.0731	−0.0894	0.9241	0.092*	
C26	0.2329 (4)	−0.12559 (16)	1.0054 (3)	0.0764 (10)	
C27	0.3634 (4)	−0.11984 (16)	1.0115 (3)	0.0863 (11)	
H27	0.4150	−0.1450	1.0591	0.104*	
C28	0.4159 (3)	−0.07660 (16)	0.9467 (3)	0.0872 (11)	
H28	0.5032	−0.0737	0.9519	0.105*	
C29	0.0443 (5)	−0.17245 (17)	1.0621 (4)	0.1289 (17)	
H29A	0.0103	−0.1368	1.0903	0.193*	
H29B	0.0216	−0.2044	1.1107	0.193*	
H29C	0.0109	−0.1791	0.9811	0.193*	
C30	0.2540 (4)	−0.20578 (17)	1.1510 (3)	0.1166 (15)	
H30A	0.3060	−0.2304	1.1075	0.175*	
H30B	0.1996	−0.2296	1.1935	0.175*	
H30C	0.3063	−0.1823	1.2064	0.175*	

### Atomic displacement parameters (Å^2^)

**Table:**

	*U*^11^	*U*^22^	*U*^33^	*U*^12^	*U*^13^	*U*^23^
S1	0.0717 (6)	0.0764 (7)	0.0644 (6)	0.0111 (5)	−0.0005 (5)	0.0060 (5)
S2	0.0801 (6)	0.0720 (6)	0.0704 (6)	0.0154 (5)	−0.0128 (5)	−0.0120 (5)
N1	0.0643 (18)	0.0658 (19)	0.0583 (17)	0.0104 (15)	0.0060 (14)	0.0049 (15)
N2	0.0727 (19)	0.064 (2)	0.073 (2)	0.0052 (16)	0.0010 (16)	0.0112 (16)
N3	0.096 (2)	0.060 (2)	0.081 (2)	0.0147 (17)	0.0124 (18)	0.0007 (17)
N4	0.114 (3)	0.083 (3)	0.105 (3)	−0.015 (2)	0.041 (2)	0.003 (2)
C1	0.063 (2)	0.059 (2)	0.053 (2)	0.0074 (17)	0.0034 (17)	0.0004 (17)
C2	0.073 (2)	0.073 (3)	0.064 (2)	0.0103 (19)	0.001 (2)	−0.0028 (19)
C3	0.100 (3)	0.075 (3)	0.064 (2)	0.015 (2)	−0.003 (2)	−0.0136 (19)
C4	0.096 (3)	0.085 (3)	0.066 (3)	0.011 (2)	−0.020 (2)	−0.012 (2)
C5	0.069 (2)	0.078 (3)	0.073 (2)	0.0173 (19)	−0.008 (2)	0.001 (2)
C6	0.064 (2)	0.062 (2)	0.048 (2)	0.0032 (18)	0.0043 (18)	0.0001 (17)
C7	0.065 (2)	0.075 (3)	0.057 (2)	0.0060 (19)	0.0044 (18)	0.0046 (19)
C8	0.062 (2)	0.060 (2)	0.062 (2)	0.0096 (17)	0.0117 (18)	0.0079 (18)
C9	0.082 (2)	0.060 (2)	0.061 (2)	0.0090 (18)	0.009 (2)	0.0105 (19)
C10	0.087 (2)	0.067 (3)	0.057 (2)	0.006 (2)	0.0074 (19)	0.0074 (19)
C11	0.058 (2)	0.056 (2)	0.077 (3)	0.0023 (17)	0.012 (2)	0.004 (2)
C12	0.076 (2)	0.059 (2)	0.079 (3)	0.0150 (18)	0.000 (2)	0.010 (2)
C13	0.078 (2)	0.071 (3)	0.066 (2)	0.015 (2)	−0.0003 (19)	0.008 (2)
C14	0.106 (3)	0.081 (3)	0.124 (3)	0.030 (2)	0.002 (3)	−0.010 (3)
C15	0.149 (4)	0.072 (3)	0.095 (3)	0.012 (2)	0.038 (3)	−0.002 (2)
C16	0.061 (2)	0.064 (2)	0.059 (2)	0.0001 (19)	−0.0057 (17)	0.0063 (17)
C17	0.080 (3)	0.067 (3)	0.092 (3)	0.003 (2)	0.003 (2)	0.010 (2)
C18	0.079 (3)	0.090 (3)	0.128 (4)	0.003 (2)	0.021 (3)	0.037 (3)
C19	0.074 (3)	0.116 (4)	0.133 (4)	0.012 (3)	0.029 (3)	0.034 (3)
C20	0.081 (3)	0.089 (3)	0.116 (3)	0.019 (2)	0.021 (3)	0.029 (2)
C21	0.061 (2)	0.070 (3)	0.071 (2)	0.006 (2)	0.0053 (19)	0.0096 (19)
C22	0.066 (2)	0.075 (3)	0.084 (3)	0.005 (2)	0.006 (2)	0.006 (2)
C23	0.059 (2)	0.065 (3)	0.073 (2)	0.0095 (19)	0.008 (2)	0.0055 (18)
C24	0.072 (3)	0.067 (3)	0.072 (2)	0.009 (2)	0.001 (2)	−0.0030 (19)
C25	0.073 (3)	0.077 (3)	0.081 (3)	−0.006 (2)	0.010 (2)	−0.009 (2)
C26	0.095 (3)	0.062 (3)	0.077 (3)	−0.002 (2)	0.028 (2)	−0.006 (2)
C27	0.080 (3)	0.078 (3)	0.103 (3)	0.020 (2)	0.020 (2)	0.029 (2)
C28	0.067 (2)	0.085 (3)	0.112 (3)	0.018 (2)	0.020 (2)	0.023 (2)
C29	0.145 (5)	0.101 (4)	0.151 (4)	−0.040 (3)	0.070 (4)	−0.015 (3)
C30	0.175 (5)	0.084 (3)	0.095 (3)	−0.006 (3)	0.034 (3)	0.015 (3)

### Geometric parameters (Å, °)

**Table:**

S1—C16	1.777 (3)	C14—H14A	0.9600
S1—S2	2.0521 (12)	C14—H14B	0.9600
S2—C1	1.786 (3)	C14—H14C	0.9600
N1—C7	1.274 (3)	C15—H15A	0.9600
N1—C6	1.419 (4)	C15—H15B	0.9600
N2—C22	1.277 (4)	C15—H15C	0.9600
N2—C21	1.404 (4)	C16—C17	1.391 (4)
N3—C11	1.376 (4)	C16—C21	1.400 (4)
N3—C15	1.440 (4)	C17—C18	1.375 (4)
N3—C14	1.441 (4)	C17—H17	0.9300
N4—C26	1.370 (4)	C18—C19	1.370 (5)
N4—C29	1.429 (5)	C18—H18	0.9300
N4—C30	1.454 (4)	C19—C20	1.380 (4)
C1—C2	1.386 (4)	C19—H19	0.9300
C1—C6	1.389 (4)	C20—C21	1.393 (4)
C2—C3	1.385 (4)	C20—H20	0.9300
C2—H2	0.9300	C22—C23	1.444 (4)
C3—C4	1.373 (4)	C22—H22	0.9300
C3—H3	0.9300	C23—C28	1.385 (4)
C4—C5	1.384 (4)	C23—C24	1.390 (4)
C4—H4	0.9300	C24—C25	1.367 (4)
C5—C6	1.391 (4)	C24—H24	0.9300
C5—H5	0.9300	C25—C26	1.408 (4)
C7—C8	1.465 (4)	C25—H25	0.9300
C7—H7	0.9300	C26—C27	1.393 (4)
C8—C13	1.383 (4)	C27—C28	1.384 (4)
C8—C9	1.395 (4)	C27—H27	0.9300
C9—C10	1.370 (4)	C28—H28	0.9300
C9—H9	0.9300	C29—H29A	0.9600
C10—C11	1.399 (4)	C29—H29B	0.9600
C10—H10	0.9300	C29—H29C	0.9600
C11—C12	1.403 (4)	C30—H30A	0.9600
C12—C13	1.373 (4)	C30—H30B	0.9600
C12—H12	0.9300	C30—H30C	0.9600
C13—H13	0.9300		
			
C16—S1—S2	106.03 (12)	N3—C15—H15B	109.5
C1—S2—S1	103.05 (11)	H15A—C15—H15B	109.5
C7—N1—C6	119.7 (3)	N3—C15—H15C	109.5
C22—N2—C21	120.7 (3)	H15A—C15—H15C	109.5
C11—N3—C15	120.7 (3)	H15B—C15—H15C	109.5
C11—N3—C14	120.5 (3)	C17—C16—C21	120.2 (3)
C15—N3—C14	118.3 (3)	C17—C16—S1	125.3 (3)
C26—N4—C29	120.0 (4)	C21—C16—S1	114.4 (3)
C26—N4—C30	121.2 (4)	C18—C17—C16	120.1 (3)
C29—N4—C30	118.6 (3)	C18—C17—H17	120.0
C2—C1—C6	120.0 (3)	C16—C17—H17	120.0
C2—C1—S2	122.4 (3)	C19—C18—C17	120.3 (4)
C6—C1—S2	117.6 (2)	C19—C18—H18	119.9
C3—C2—C1	120.5 (3)	C17—C18—H18	119.9
C3—C2—H2	119.8	C18—C19—C20	120.3 (4)
C1—C2—H2	119.8	C18—C19—H19	119.8
C4—C3—C2	119.8 (3)	C20—C19—H19	119.8
C4—C3—H3	120.1	C19—C20—C21	120.8 (4)
C2—C3—H3	120.1	C19—C20—H20	119.6
C3—C4—C5	119.9 (3)	C21—C20—H20	119.6
C3—C4—H4	120.0	C20—C21—C16	118.2 (3)
C5—C4—H4	120.0	C20—C21—N2	125.8 (3)
C4—C5—C6	120.8 (3)	C16—C21—N2	115.9 (3)
C4—C5—H5	119.6	N2—C22—C23	123.4 (3)
C6—C5—H5	119.6	N2—C22—H22	118.3
C1—C6—C5	118.8 (3)	C23—C22—H22	118.3
C1—C6—N1	117.7 (3)	C28—C23—C24	116.2 (3)
C5—C6—N1	123.4 (3)	C28—C23—C22	121.2 (3)
N1—C7—C8	124.3 (3)	C24—C23—C22	122.6 (3)
N1—C7—H7	117.9	C25—C24—C23	121.9 (3)
C8—C7—H7	117.9	C25—C24—H24	119.1
C13—C8—C9	116.7 (3)	C23—C24—H24	119.1
C13—C8—C7	120.7 (3)	C24—C25—C26	121.6 (3)
C9—C8—C7	122.5 (3)	C24—C25—H25	119.2
C10—C9—C8	121.6 (3)	C26—C25—H25	119.2
C10—C9—H9	119.2	N4—C26—C27	121.1 (4)
C8—C9—H9	119.2	N4—C26—C25	121.8 (4)
C9—C10—C11	121.6 (3)	C27—C26—C25	117.1 (3)
C9—C10—H10	119.2	C28—C27—C26	119.9 (4)
C11—C10—H10	119.2	C28—C27—H27	120.1
N3—C11—C10	121.7 (3)	C26—C27—H27	120.1
N3—C11—C12	121.7 (3)	C27—C28—C23	123.3 (3)
C10—C11—C12	116.6 (3)	C27—C28—H28	118.3
C13—C12—C11	120.9 (3)	C23—C28—H28	118.3
C13—C12—H12	119.6	N4—C29—H29A	109.5
C11—C12—H12	119.6	N4—C29—H29B	109.5
C12—C13—C8	122.4 (3)	H29A—C29—H29B	109.5
C12—C13—H13	118.8	N4—C29—H29C	109.5
C8—C13—H13	118.8	H29A—C29—H29C	109.5
N3—C14—H14A	109.5	H29B—C29—H29C	109.5
N3—C14—H14B	109.5	N4—C30—H30A	109.5
H14A—C14—H14B	109.5	N4—C30—H30B	109.5
N3—C14—H14C	109.5	H30A—C30—H30B	109.5
H14A—C14—H14C	109.5	N4—C30—H30C	109.5
H14B—C14—H14C	109.5	H30A—C30—H30C	109.5
N3—C15—H15A	109.5	H30B—C30—H30C	109.5
